# Stability of p53 Homologs

**DOI:** 10.1371/journal.pone.0047889

**Published:** 2012-10-24

**Authors:** Tobias Brandt, Joel L. Kaar, Alan R. Fersht, Dmitry B. Veprintsev

**Affiliations:** 1 MRC Centre for Protein Engineering, Cambridge, United Kingdom; 2 MRC Laboratory of Molecular Biology, Cambridge, United Kingdom; Cardiff University, United Kingdom

## Abstract

Most proteins have not evolved for maximal thermal stability. Some are only marginally stable, as for example, the DNA-binding domains of p53 and its homologs, whose kinetic and thermodynamic stabilities are strongly correlated. Here, we applied high-throughput methods using a real-time PCR thermocycler to study the stability of several full-length orthologs and paralogs of the p53 family of transcription factors, which have diverse functions, ranging from tumour suppression to control of developmental processes. From isothermal denaturation fluorimetry and differential scanning fluorimetry, we found that full-length proteins showed the same correlation between kinetic and thermodynamic stability as their isolated DNA-binding domains. The stabilities of the full-length p53 orthologs were marginal and correlated with the temperature of their organism, paralleling the stability of the isolated DNA-binding domains. Additionally, the paralogs p63 and p73 were significantly more stable and long-lived than p53. The short half-life of p53 orthologs and the greater persistence of the paralogs may be biologically relevant.

## Introduction

The role of stability in the evolution of proteins is intriguing. Many enzymes from thermophilic organisms are less active than those from mesophilic organisms when assayed at mesophilic temperatures. On the other hand, early protein engineering studies showed that the stability of proteins can be increased by mutation without losing activity. It is generally assumed that the stability of proteins is evolutionally adapted to the temperature of their environment. For proteins that have evolved to be unstable, instability offers a mechanism by which cells can tightly control intracellular protein concentrations, which can be crucial for cell survival [1], [2], [3], [4], [5].

Members of the p53 family of transcription factors have fundamental but distinct roles. p53 is at the centre of a tumour suppressor network whereas p63 and p73 have mainly developmental functions [6], [7]. Protein stability plays a major role in regulating cellular levels of p53. Several lines of evidence suggest that p53 may have evolved to be relatively unstable with its low melting temperature of about 45°C [8], [9], [10], [11], [12]. Correspondingly, many tumours are linked to the malfunction of p53 caused by destabilising mutations [13], [14].

The *in vitro* stability of the DNA-binding domain (DBD) has been extensively characterised for p53 [10], several p53 orthologs [15] as well as for human p63 and p73 [8], [16], [17]. Thermal and chemical denaturation studies show that the thermodynamic stability of the p53 DBD, and as a result the ability to bind DNA, is severely compromised by a significant fraction of the tumour-associated mutations [10], [11]. Further, the rate of denaturation of the human p53 DBD at 37°C is correlated to the thermal stability of several mutants studied [18], a correlation also observed for DBDs of several orthologs of p53 [15]. Protein engineering studies have shown that the stability of p53DBD can be increased by mutagenesis [19], [20], [21] and is, therefore, not constrained by sequence. The thermodynamic stability of the DBD of p53 orthologs also correlates with the body temperature for higher animals and especially for mammals, but not for arthropods or nematodes [15]. In contrast to p53, isolated DBDs of p63 and p73 are significantly more stable than p53 [8], [16], [17]. Taken together, existing data suggest that stability of p53DBD is evolutionary fine-tuned to ensure that p53 is just stable enough to function at human body temperature.

Full-length proteins of the p53 family contain additional structured domains such as the tetramerisation domain or, in the case of the α-isoforms of p63 and p73, a sterile-alpha motif domain. Intrinsically disordered domains, flanking the DBD at its amino and carboxy termini, may also affect overall protein stability [22]. These additional domains are far less conserved than the DBDs and thus may affect stability differently. It is, therefore, important to study the stability of full-length proteins as well as stability of DBDs in the context of the full-length proteins.

Here, we examined the thermodynamic and kinetic stability of full-length proteins of the p53 family using differential scanning fluorimetry (DSF) and isothermal denaturation fluorimetry (ITDF). Specifically, we have compared human p53 with orthologous p53 from evolutionarily close and distant organisms as well as with its paralogs p63 and p73. DSF and ITDF involve monitoring the unfolding of a protein in the presence of an exogenous fluorescent dye reporter, which fluoresces strongly upon binding to hydrophobic sites of a protein. In DSF, protein unfolding is followed while the temperature is continuously increased [23], [24]. Conversely, in ITDF, the unfolding of a protein is followed at constant temperature over time, yielding kinetic data and the protein’s half-life (*t*_1/2_) [25], [26], [27]. A high-throughput, real-time PCR thermocycler with very low demands on protein amounts and excellent temperature control was used for DSF and ITDF, permitting high accuracy melting temperature and half-life measurements.

## Results and Discussion

### Evolution of Protein Stability within the p53 Family

The domain structure of the p53 family is shown in Figure S1. To study the stability of the p53 family, we expressed and purified full-length p53 from frog (*Xenopus laevis*, Xlp53), fruit fly (*Drosophila melanogaster*, Dmp53), mouse (*Mus musculus*, Mmp53), and zebrafish (*Danio rerio*, Drp53). We also expressed and purified full-length human p53 (Hsp53) as well as a super-stable variant of full-length human p53 (QM-Hsp53) [21]. In addition to full-length p53, isolated core domains of wild-type and stable human p53 (Hsp53DBD and QM-Hsp53DBD, residues 94–312) and human p53 deletion constructs missing either the C-terminal domain (QM-Hsp53NCT, residues 1–355), the N-terminal domain (QM-Hsp53CTC, residues 94–393) or both (QM-Hsp53CT, residues 94–355) were expressed and purified. Additionally, we purified the human isoforms ΔNp63α, ΔNp63β, ΔNp63γ, ΔNp73α, ΔNp73β, TAp73β, and TAp73γ as well as constructs of the p63 core domain (p63DBD, residues 115–351) and the p73 core domain (p73DBD, residues 109–312).

#### Kinetics of denaturation

The process of denaturation and subsequent aggregation of the p53DBD is described by scheme 1 [18], [28], [29]:
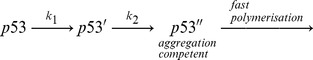



The denaturation and aggregation of the human protein follows simple sequential first-order kinetics but under certain conditions *k*_1_ is rate determining and *k*_2_ is not observed when monitored by probes that effectively measure loss of native protein although methods that monitor aggregate formation conform to lag kinetics. The simplest kinetics for the exponential phase follow equation 1, being composed of *k*_obs_ and a linear drift term from the subsequent aggregation process.
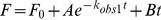
(1)


**Figure 1:**
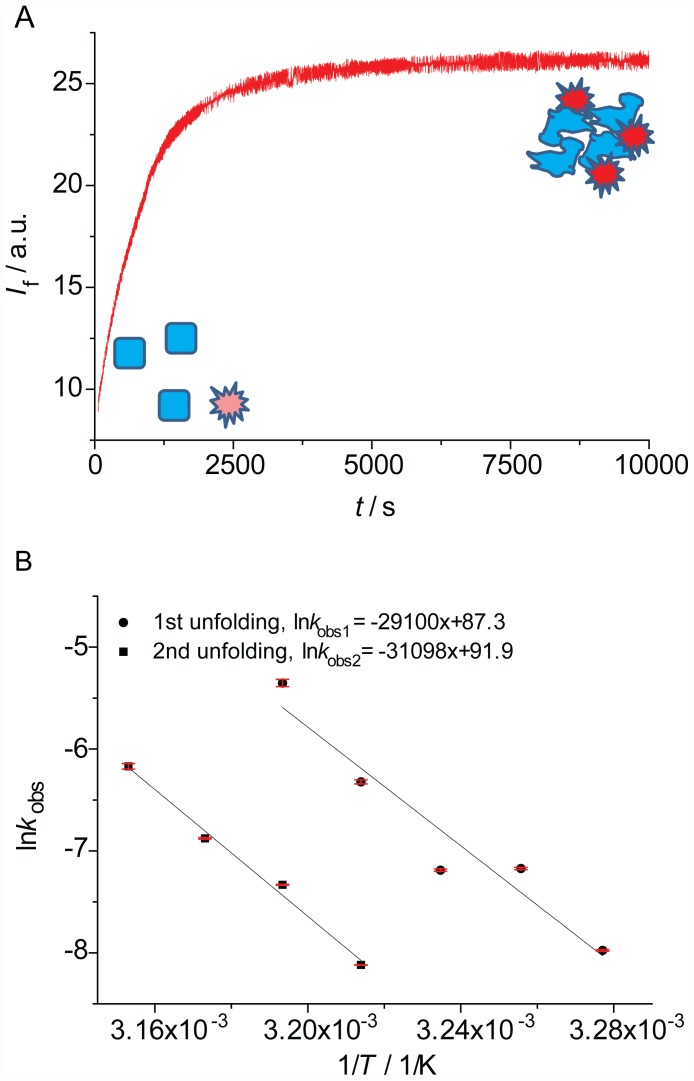
ITDF results. A: A typical denaturation curve. Initially (*t* = 0) well-folded proteins (squares, blue) and fluorescent dye (star, light red) are mixed, and the fluorescence is low. Over time the protein denatures (undefined shape, blue) and its exposed hydrophobic core binds to the dye (stars, bright red), leading to a fluorescence increase which is monitored. B: Examples of Arrhenius plots for Mmp53 of the unfolding of the quaternary structure (*k*_1_, circles) and the core domain (*k*_2_, squares).

For several members of the p53 family, we measured the kinetic stability using ITDF. A typical denaturation curve is shown in Figure 1A. The apparent exponential increase in fluorescence is directly proportional to the increase in denatured species. In order to measure accurately the unfolding kinetics, the exponential phase was followed for at least five half-lives and was analysed in isolation from non-exponential intensity changes due to equilibration or precipitation.

In most cases data were fitted to a single-exponential model (equation 1) to yield the unfolding rate constant *k*_obs1_. However, in the case of mammalian full-length p53 family members (Hs53, QM-Hp53 and Mmp53), we detected the additional, faster unfolding event (*k*_2_), which at certain temperatures required a double-exponential model (equation 2).
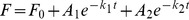
(2)


In both models, *F* denotes the measured fluorescence, *F*_0_ the intrinsic fluorescence of the sample as measured at the start of the analysis, *A* the amplitude of fluorescence change, and *k* the observed rate constant of denaturation. In traces with two unfolding events, the second transition was modelled either as a slow transition, using a linear drift term (*Bt*), or as a second exponential term. *t*_1/2_ was calculated from *t*_1/2_ = 0.6931/*k*. As *k*_1_ and *k*_2_ are interchangeable in equation 2, one cannot automatically assign the faster phase to *k*_1_ and the slower to *k*_2_ in scheme 1, or vice versa.

For all the proteins studied, we collected kinetic stability data at several temperatures. This allowed the use of Arrhenius plots (Figure 1B) to calculate the temperature at which *t*_1/2_ is 15 min (*T*_15_, Table 1). This time was, for all the proteins studied, well within the linear range of the Arrhenius plots covered by the measurements.

**Table 1:** Thermal and kinetic stability data for p53 family members.

Protein	*T*_15_/°C (*t*_1/2_ = 15 min)^b^	*E*_A_/kJ/mol^b^	*T*_m_/°C ± SD (DSF)	*T*_m_/°C ± SD (DSC)	*T*_env_/°C^a^
Hsp53	38.0 (36.5)	373 (165)	46.2±0.1	–	36.8
QM-p53	43.6 (38.4)	491 (480)	51.9±0.2	52.1±0.1	
Hsp53DBD	38.2	478	45.6±0.1	–	
QM-p53DBD	44.4	594	50.2±0.1	–	
QMp53NCT	43.3	335	–	–	
QMp53CT	43.3	361	–	–	
QMp53CTC	43.4	349	–	–	
p63DBD	–	–	61.5±0.0	62.0±0.2	
ΔNp63α	–	–	56.7±0.1	–	
ΔNp63β	44.8	276	55.9±0.0	56.8±0.3	
ΔNp63γ	47.5	434	57.3±0.0	57.8	
ΔNp73α	–	–	50.1±0.2	–	
ΔNp73β	41.0	459	48.4±0.0	48.9±0.3	
p73DBD	41.0	254	49.4±0.1	–	
TAp73β	41.1	295	49.4±0.2	–	
TAp73γ	–	–	48.9±0.4	–	
Dmp53	33.0	222	44.6±0.3	– (49.4^c^)	26–31 [43]
Drp53	*t*_1/2_<60 s^d^	–	40.5±0.0	39.8 (41.4^c^)	22–30 [44]
Mmp53	40.9 (34.8)	259 (242)	46.6±0.1	46.6±0.4 (45.9^c^)	36.9
Xlp53	*t*_1/2_<60 s^d^	–	34.0±0.4	35.0±0.4 (38.0^c^)	19–24 [45]

a For the homeothermic organisms (human and mouse) the body temperature is given. For poikilothermic organisms (fruit fly, frog and zebrafish) optimal conditions for development are stated.

b Values for kinetic denaturation (*T*_15_ and *E*_A_) of the core domain (*k*_1_) and in parenthesis for the faster unfolding event (*k*_2_) are given.

c DBD only [15].

d No Arrhenius plot obtained. Proteins unstable at 37°C.

To confirm the accuracy of ITDF measurements, denaturation kinetics were also measured by monitoring intrinsic protein fluorescence. Specifically, the fluorescence of tyrosine residues of QM-Hsp53 was followed using a cuvette-based fluorimeter (Figure S2). This method utilises the decrease in fluorescence intensity of tyrosine residues within the p53DBD upon unfolding [18]. It is predominantly sensitive to the unfolding of the DBD as it contains 8 out of the 9 tyrosine residues in p53. The resulting Arrhenius plot was very similar to the one obtained by ITDF (Figure S2), suggesting that increased fluorescence of the exogenous fluorescent dye reporter Sypro Orange reflects the loss of native protein.

Similar to the isolated DBD, full-length Hsp53 was significantly less stable than QM-Hsp53. For Hsp53 and QM-Hsp53, the rate constants determined for the slower unfolding event (*k*_1_) were very similar to those obtained for their respective isolated DBDs.

**Figure 2:**
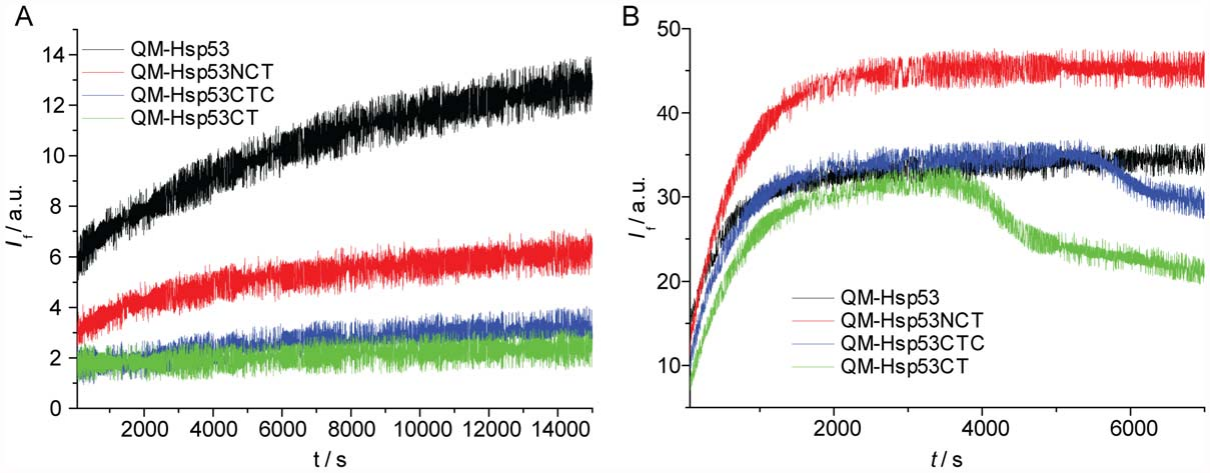
ITDF results for QM-Hsp53 constructs. A: Measurement at 38°C, full-length QM-Hsp53 in black and a construct missing the C-terminus (QM-Hsp53NCT, red) exhibit the second, faster unfolding event. Constructs missing the N-terminus (QM-Hsp53CTC, blue; QM-Hsp53CT, green) do not exhibit any significant unfolding. B: Measurement at 45°C using the same colour coding as in ‘A’. Fluorescence drops are only seen for constructs missing the N-terminus of p53.

In the analysis of the full-length proteins, the second, faster event was resolved at lower temperatures. In order to attribute it to an unfolding process of p53, we analysed the concentration dependence of QM-Hsp53 at 38°C (Table 2). The half-life of the fast transition decreased with increasing protein concentrations. Further, denaturation traces of p53 domain-deletion constructs lacking the N-terminal domain (QM-Hsp53DBD, QM-Hsp53CT, QM-Hsp53CTC, Figure 2A) did not exhibit a second unfolding event at lower temperatures, whereas the kinetics of unfolding of the DBD (*k*_1_) were very similar in these constructs (Table 1). In contrast, QM-p53NCT, which included the acidic N-terminal domain, showed this second, faster unfolding event but with much smaller amplitude than for the full-length protein. The slope of the Arrhenius plot (Figures 1B and S2) was similar for both unfolding events, suggesting similar activation energies. A possible explanation for these observations is that the acidic N-terminus of p53 interacts transiently and unspecifically with the basic DBD and C-terminal domains of other p53 molecules [22]. As the concentration of protein in these experiments (15–60 µM) was much higher than the dissociation constant of the p53 tetramer (ca. 20 nM) [30], such interactions are likely to involve different tetramers. It remains to be seen if this observation has physiological relevance.

**Table 2:** Kinetic stability of QM-Hsp53. Effect of ligands and protein concentration.

T/°C	c/µM	ligand	t_1/2_/s	n	SD/s
45	15	–	390	4	16
	15	2 mg/mL heparin	780	4	28
	15	5 µM DNA	990	4	46
38	15	–	4800	3	74
	30	–	3700	4	127
	60	–	2400	4	115

Additionally, the precipitation of protein aggregates, characterised by a drop in fluorescence intensity, depended on the p53 construct used. Earlier studies have shown that the p53DBD readily aggregates under different denaturing conditions, thereby making the process of denaturation irreversible [10], [18], [28], [31], [32]. Here, aggregation occurred earlier for constructs without the N-terminal domain (QM-Hsp53DBD, QM-Hsp53CT and QM-Hsp53CTC), but was not detected within the time frame examined for QM-p53NCT and full-length p53 (Hsp53 and QM-Hsp53, Figure 2B). This suggests that the N-terminal domain is also important in preventing p53 aggregation.

The p53 paralogs ΔNp73β, p73DBD and especially ΔNp63β and ΔNp63γ were kinetically more stable than Hsp53. Interestingly, only very low critical concentrations of small aggregates triggered precipitation after unfolding for all paralogs. For p63DBD this effect prevented unfolding analysis. However, despite this, it was possible to ascertain that the protein was unstable (*t*_1/2_<60 s) at 57°C, but stable below 45°C. Additionally, in contrast to Hsp53, the p73DBD was kinetically less stable than naturally occurring isoforms. Finally, we found that the kinetic stability of TAp73β is almost identical to that of ΔNp73β (see Table 1). However, no precipitation was observed within the time frame examined for TAp73β, the only paralog analysed with a full-length N-terminus. Similarly to p53, this suggests an involvement of the N-terminal TA-domain in aggregation prevention.

The murine ortholog of Hsp53, Mmp53, also displayed two unfolding events. Both rate constants were very similar to those of Hsp53. Dmp53, Drp53 and Xlp53 were kinetically less stable than mammalian full-length p53, but their propensity to denature at or around ambient temperature significantly hampered accurate stability measurements. Drp53 and Xlp53 in particular were unstable at 32°C, which, due to temperature control limitations, was the lowest temperature at which stability could be measured.

#### Specific and non-specific binding prolongs p53 half-life

After initial stability measurements, the effect of specific and non-specific binding partners that modulate the kinetic stability of p53 *in vivo*, thereby contributing to longer protein activity, was examined. It has been shown previously that the thermodynamic stability of Hsp53DBD is increased by the addition of ligands like heparin [11] and drug-like small molecules [33]. Additionally, Hsp53DBD and Hsp53 have been shown to be more stable under pressure denaturation conditions if bound to the specific p53 DNA response element [34]. Furthermore, the protein stability *in vitro* is affected by the buffer composition.

**Figure 3:**
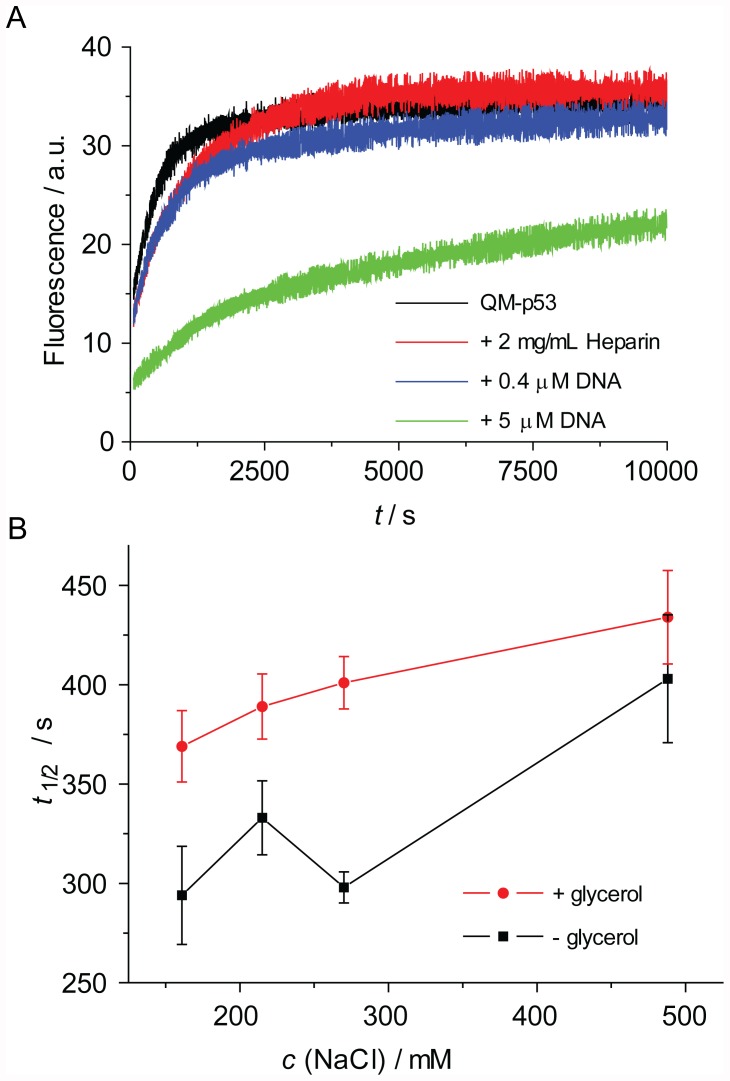
Kinetic stabilisation of p53. A: Raw data for QM-p53 alone (black), with added heparin (red) and with added DNA (0.4 µM, blue; 5 µM, green). B: Comparison of half-lives of QM-p53 at different NaCl concentration without (black) and with 10% glycerol (red).

The addition of heparin (2 mg/mL) approximately doubled the half-life of QM-Hsp53 (Table 2, Figure 3A). Even more pronounced was the stabilisation of QM-Hsp53 upon addition of 5 µM DNA, containing a p53 response element (*K*_d_ = 120 nM [35]). In contrast to free QM-Hsp53 at 45°C, the data for DNA-bound protein were best described by a double-exponential model, indicating a significant stabilisation of full-length p53 upon binding of DNA. Taken together, our results confirm that the kinetic stability of full-length p53 can be extended by protein-ligand interactions.

Additionally, we determined the effect of the buffer additives glycerol and sodium chloride on the kinetic stability of QM-Hsp53 (Figure 3B). In the presence of 10% glycerol, QM-Hsp53 was stabilised by about 20%. Similarly, increasing sodium chloride concentration lengthened the *t*_1/2_ of QM-Hsp53. The small differences induced by non-specific additives can be reliably detected using ITDF. Identification of optimal buffer conditions is of importance for protein formulations, such as for, among others, vaccines and biological therapeutics.

### Correlation between Kinetic and Thermodynamic Stability

**Figure 4:**
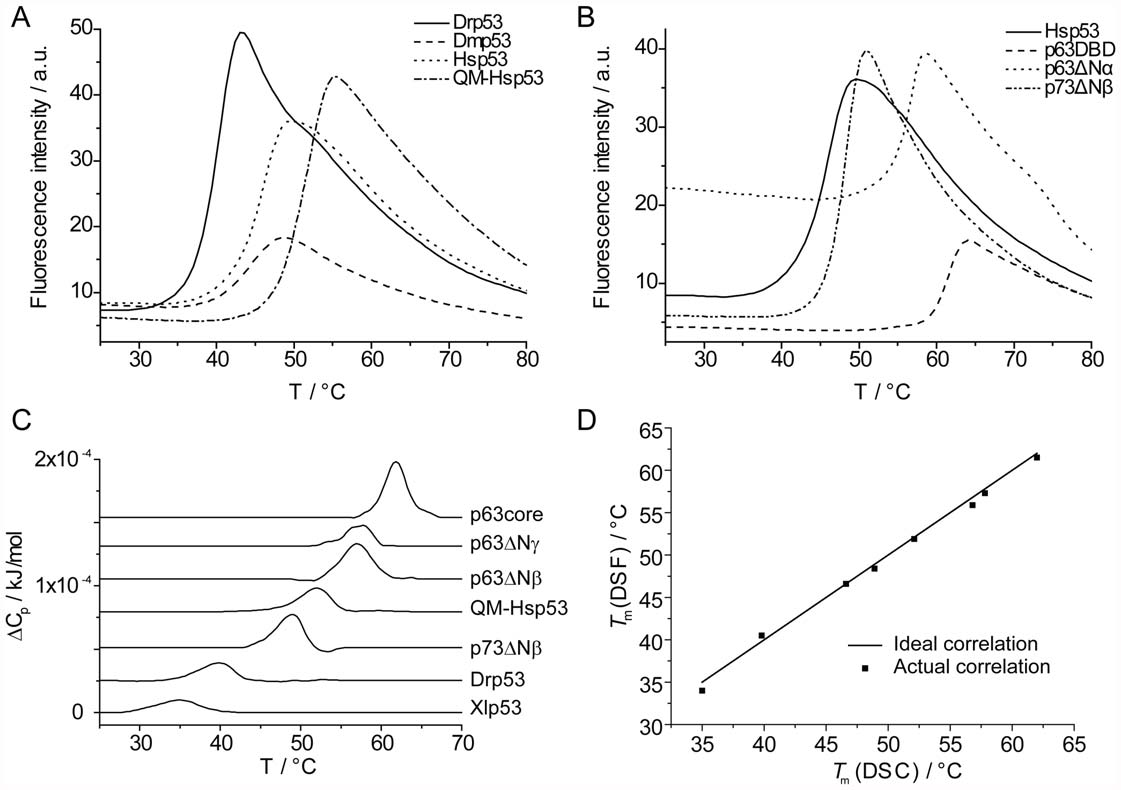
Thermodynamic stability determination. A: Examples of raw data melting curves for p53 orthologs derived from DSF experiments. *T*_m_ is defined as the maximum of the first derivative. B: DSF melting curves for p53 paralogs. C: Stacked view of DSC melting curves. D: Correlation of melting temperatures derived by DSF and DSC.

Mutants of Hsp53DBD exhibit a correlation between kinetic and thermodynamic stability [18]. To determine if there is such a correlation among full-length p53 family members that contain considerable amino acid differences, we measured apparent *T*_m_ values (Table 1) by following the unfolding process using DSF (Figure 4A, B). Strictly speaking, p53 and its homologs denature irreversibly and the measured apparent *T*_m_ varies with the heating rate. However, if the heating rate is sufficiently fast, the measured *T*_m_ approximates to its true value because equilibration, which in turn is fast compared with the heating rate, is faster than the irreversible process [33]. We used DSC experiments as controls and found that results of DSF and DSC were in good agreement (Figure 4C and D). The thermal stability of p53 orthologs mirrored their kinetic stability. The *T*_m_ of Hsp53 and Mmp53 were very similar and the thermodynamic stability of the remaining p53 orthologs (Dmp53, Drp53 and Xlp53) was significantly lower. In contrast to Hsp53, the full-length proteins Dmp53 and Xlp53 were 4–5°C less stable than their respective, isolated DBDs.

**Figure 5:**
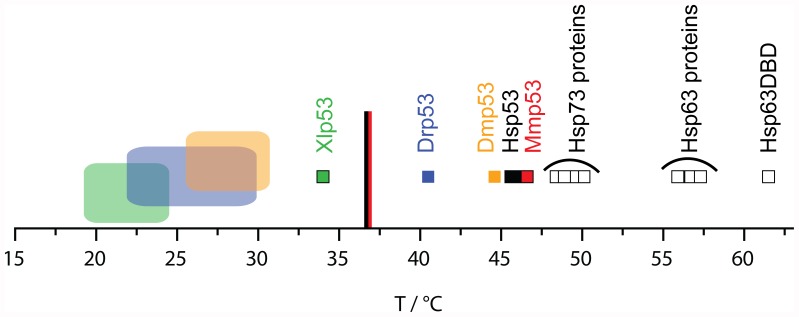
Overview of protein melting temperatures (small squares) within the p53 family. p53 orthologs (filled squares; Hsp53 in black, Mmp53 in red, Dmp53 in orange, Drp53 in blue and Xlp53 in green) and p53 paralogs (hollow squares) are shown. In corresponding colours body temperatures of homeothermic organisms are indicated as lines and optimal development conditions of poikilothermic organisms are illustrated as large rectangles.

The stability data of several full-length p53 orthologs supported a model that p53 has evolved to have only marginal thermodynamically and kinetically stability under its different organismal environmental conditions. The lower the body temperature (for homeothermic species) or optimal development temperature (for poikilothermic species) of an organism, the less stable is the p53 of this organism (Table 1, Figure 5). A similar trend has been reported for the isolated DBDs of homeothermic species, but was not as pronounced for those of poikilothermic species [15]. We conclude that the less conserved N-and C-terminal domains of Dmp53 and to a lesser extent also of Drp53 and Xlp53 might have evolved to destabilise the protein and adapt to the pressure of lower environmental temperatures, while the stability of the DBD changed to a lesser degree. Further, the margin between melting temperature and environmental temperature was found to be slightly smaller in organisms that regulate their body temperature than in those that do not.

The ΔN-isoforms of p63 studied, α, β and γ, were about 10°C more stable than p53 but about 5°C less stable than the p63DBD in isolation in terms of *T*_m_. Thus, for p63, analysis of the stability of the DBD alone is not representative of that of the full-length protein. Small differences in stability of p63 were due to the isoform-specific C-termini, with the β-isoform being the least and the γ-isoform being the most stable protein. All of the p73 isoforms studied were about 3–4°C more stable than Hsp53 and isoform-specific differences were small. For previously studied proteins QM-Hsp53, QM-Hsp53DBD and p63DBD [8], [16], we found 1–2°C higher *T*_m_ values, which was presumably due to the stabilising effect of the 10% glycerol in our experiments.

A possible reason for greater stability of p63 and p73 relative to p53 may lie in their respective cellular roles. As a tumour suppressor p53 may cause cell-cycle arrest or apoptosis. It is thought that the stability of p53 is compromised in order to enable tight control of its abundance in healthy cells [36]. In contrast, both p63 and p73 have evolved to be kinetically and thermodynamically more stable than p53, making them less susceptible to thermal denaturation and unfolding, which is beneficial to their involvement in long-term developmental processes. In addition, deletion of p63 and p73 leads to lethality or strong developmental defects [37], [38]. In contrast to p53, p63 and p73 may, therefore, need to be thermodynamically “protected” from destabilising mutations.

**Figure 6:**
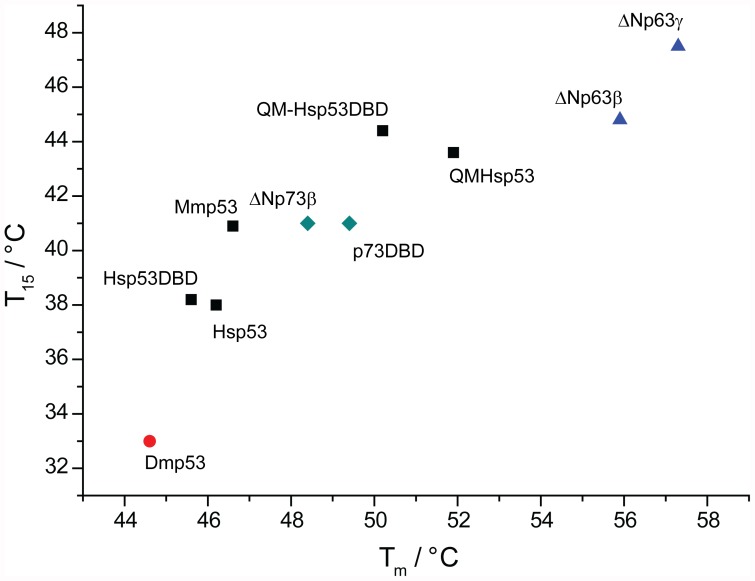
Correlation between kinetic (*T*_15_, unfolding of DBD) and thermodynamic (*T*_m_, DSF) stability data. Mammalian p53 proteins studied are represented by black squares, human p63 isoforms by blue triangles, human p73 constructs by green diamonds and Dmp53 by a red circle.

Generally, the thermodynamic stability (*T*_m_) measured for the p53 family members that were studied, correlated well with their kinetic stability (*T*_15_, unfolding of the DBD) (Figure 6). However, the correlation was not perfectly linear. Interestingly, the DBDs of full-length Hsp53 and QM-Hsp53 were kinetically slightly less stable, but thermodynamically more stable than their respective isolated DBDs. Noticeably, the p63 isoforms studied and Dmp53 were kinetically relatively less stable than Hsp53, which was evident from the larger difference between their respective *T*_m_ and *T*_15_ values.

### Conclusions

Using a combined approach of ITDF and DSF we obtained a comprehensive picture of the stability of full-length proteins from the p53 family. Most significantly, ITDF and DSF allowed the study of protein unfolding processes, including that of full-length multi-domain proteins. In addition, due to the minimal sample amount requirements of these methods, it was possible to determine the stability of a range of proteins, which could not be expressed in sufficient quantity for analysis by classical methods. The parameters obtained by ITDF and DSF correlated very well. In addition, DSF and ITDF can be used for characterisation of the effects of additives such as small molecules on the protein stability of the p53 family. As an example, we show that stabilisation of the DNA-binding domain by optimisation of buffer conditions or by binding of a specific ligand (cognate DNA oligonucleotide) prolongs the half-life of the full-length human p53.

Interestingly, we found that, despite strong sequence conservation, the stability of the proteins studied varied considerably. Reflecting the stability of the isolated DNA-binding domains, the stability of the full-length p53 orthologs were found to be marginal and constrained in a very narrow range. As for the DNA-binding domain of homoeothermic organisms [15], protein stability correlated with the respective temperature of their host environment for both poikilothermic and homoeothermic organisms (Figure 5). In most cases, the stability of the full-length protein was the same as of its DNA-binding domain. Full-length proteins, similar to their isolated DNA-binding domains, also showed the same correlation between kinetic and thermodynamic stability, suggesting that the DNA-binding domain controls their stability.

While p53 from all analysed organisms appeared to have evolved to be marginally stable, p63 and p73 are significantly more stable and long-lived. It is interesting to note that unlike for the other proteins studied, the stability of several full-length isoforms of p63 was significantly decreased relative to the isolated p63 DNA-binding domain. This difference suggests that other domains can contribute to the stability control. The different stability of the isoforms may be related to *in vivo* function.

In summary, full-length p53 family members behaved similarly to the isolated DNA-binding core domains in respect to their stability. This correlation emphasises that observations made for isolated core domains of p53 are also valid for the full-length proteins. Exceptions were observed for evolutionary more distant homologs such as human p63 or Dmp53, in which other domains contribute significantly to overall protein stability. However, in the case of human p53, the correlation reinforces the validity of the concept that stabilisation of the core domain of mutant p53 by specific ligands is a viable therapeutic strategy.

## Materials and Methods

### Gene Cloning

For human full-length p53 we used WT-Hsp53 protein and a super-stable mutant, which has four mutations in the core domain (QM-Hsp53): M133L/V203A/N239Y/N268D [21], [39]. A plasmid encoding Mmp53 was kindly provided by Geoffrey Wahl. We amplified Dmp53 from a cDNA library kindly provided by Simon Bullock. Sequences encoding other studied proteins were amplified from clones provided by the MGC collection (distributed via Geneservice). For p63γ isoforms, parts of the gene were amplified from a genomic DNA library (Geneservice). Additionally, we made a p73-DBD (109–312) and a p63DBD (115–351) construct. All inserts were cloned into a pET24a-HLTEV plasmid containing the N-terminal 6×His purification tag, a lipoyl domain [40] for improved solubility and a TEV-protease cleavage site.

### Protein Expression and Purification

Small scale expression screening was done as described before [30]. Large-scale expression and purification was carried out as described earlier [30], [41], [42]. All the proteins studied were overexpressed in *E. coli* BL21 or B834 cells (Novagen) at 18°C for 16–20 h and purified using standard Ni-affinity chromatography protocols. Subsequently, the N-terminal tags were cleaved off by TEV-protease digestion. For p53 orthologs, heparin affinity chromatography was then used. The final purification step was gel filtration chromatography using a Superdex 200 16/60 preparative gel filtration column (GE Healthcare) in 225 mM NaCl, 25 mM sodium phosphate pH 7.2, 10% glycerol and 5 mM DTT. Protein purity of >95% was determined by SDS-gel electrophoresis. Samples were flash frozen in liquid nitrogen and stored at −80°C until used.

### Isothermal Denaturation Fluorimetry

The proteins studied were dialysed against buffer A (225 mM NaCl, 25 mM phosphate (pH 7.2), 10% glycerol, 5 mM DTT) at 4°C. Total sample size was 20 µL (buffer A/DMSO 10∶1). Protein and water soluble ligands were introduced in buffer A, whereas SYPRO orange (5000×, Invitrogen) was introduced in DMSO. Final protein concentrations were 15 µM. SYPRO orange was diluted to a final dilution of 25×. Heparin (sodium salt from bovine intestinal mucosa, Fluka) was used at a final concentration of 2 mg/mL. We used palindromic, self-annealing DNA (GGACATGTCCGGACATGTCC, Eurogentec) at 0.4 and 5 µM.

Fluorescence was measured with a RotorGene 6000 qPCR machine (Qiagen) using an excitation wavelength of λ_ex = _460 nm and an emission wavelength of λ_em = _610 nm. The samples were equilibrated for about 30 s, a process which can be followed because fluorescence itself is temperature dependent. Datasets were, therefore, trimmed at 60 s to cut any temperature equilibration-related artefacts and as soon as fluorescence dropped sharply due to precipitation. The data obtained had a very good signal to noise ratio, and temperature fluctuations during a run were found to be negligible. Reproducibility was good, reflected by a standard error of 6.8%, averaged over all data collected for the proteins studied. The fast acquisition of data points enabled us to reliably detect kinetic processes with half-lives down to about 100 s. Data analysis was done with ORIGIN software (Microcal) and laboratory-developed software DataFitter (http://www.mrc-lmb.cam.ac.uk/dbv/). Experiments were done at three temperatures at least and were repeated four times.

### Differential Scanning Fluorimetry

All experiments were carried out in buffer A. Sample size was 20 µL and protein concentrations of 8–14 µM were used. For Dmp53, TAp73β and TAp73γ only 3–5 µM samples could be obtained. SYPRO orange fluorescent dye (5000×, Invitrogen) was added to protein solutions and was optimised for each protein to a final dilution of 5×–50×. Melting curves were measured between 25°C and 80°C with a scan rate of 270°C/h (comparable to DSC experiments) using a RotorGene 6000 qPCR machine (Qiagen) and were carried out in quadruplicates. Data analysis was done with manufacturer supplied software.

### Differential Scanning Calorimetry

All experiments were carried out in buffer A. We used a VP-DSC (Microcal) instrument with an auto-sampler and heated the samples from 15 to 80°C at a scan rate of 125°C/h. These high scanning rates unfold the protein faster than the rate of thermal denaturation in order to measure the apparent melting temperatures, *T*_m_. 400 µL of 10 µM samples were used and experiments were repeated up to three times depending on the amount of protein available. Blank experiments with buffer were carried out as well and used as a baseline. Data analysis was done with ORIGIN software (Microcal) and the laboratory-developed software, DataFitter.

## Supporting Information

Figure S1 **p53 family domain organisation.**
(TIF)

Figure S2 **Kinetic denaturation of p53.**
(TIF)

## References

[1] DePristo MA, Weinreich DM, Hartl DL (2005) Missense meanderings in sequence space: a biophysical view of protein evolution. Nat Rev Genet 6: 678–687. 16074985 10.1038/nrg1672

[2] Somero GN (2004) Adaptation of enzymes to temperature: searching for basic “strategies”. Comp Biochem Physiol B Biochem Mol Biol 139: 321–333. 15544958 10.1016/j.cbpc.2004.05.003

[3] Tokuriki N, Tawfik DS (2009) Stability effects of mutations and protein evolvability. Curr Opin Struct Biol 19: 596–604. 19765975 10.1016/j.sbi.2009.08.003

[4] Serrano L, Day AG, Fersht AR (1993) Step-wise mutation of barnase to binase. A procedure for engineering increased stability of proteins and an experimental analysis of the evolution of protein stability. J Mol Biol 233: 305–312. 8377205 10.1006/jmbi.1993.1508

[5] Somero GN (1995) Proteins and temperature. Annu Rev Physiol 57: 43–68. 7778874 10.1146/annurev.ph.57.030195.000355

[6] Yang A, Schweitzer R, Sun D, Kaghad M, Walker N, et al (1999) p63 is essential for regenerative proliferation in limb, craniofacial and epithelial development. Nature 398: 714–718. 10227294 10.1038/19539

[7] Yang A, Walker N, Bronson R, Kaghad M, Oosterwegel M, et al (2000) p73-deficient mice have neurological, pheromonal and inflammatory defects but lack spontaneous tumours. Nature 404: 99–103. 10716451 10.1038/35003607

[8] Ang HC, Joerger AC, Mayer S, Fersht AR (2006) Effects of common cancer mutations on stability and DNA binding of full-length p53 compared with isolated core domains. J Biol Chem 281: 21934–21941. 16754663 10.1074/jbc.M604209200

[9] Bullock AN, Fersht AR (2001) Rescuing the function of mutant p53. Nat Rev Cancer 1: 68–76. 11900253 10.1038/35094077

[10] Bullock AN, Henckel J, DeDecker BS, Johnson CM, Nikolova PV, et al (1997) Thermodynamic stability of wild-type and mutant p53 core domain. Proc Natl Acad Sci U S A 94: 14338–14342. 9405613 10.1073/pnas.94.26.14338 PMC24967

[11] Bullock AN, Henckel J, Fersht AR (2000) Quantitative analysis of residual folding and DNA binding in mutant p53 core domain: definition of mutant states for rescue in cancer therapy. Oncogene 19: 1245–1256. 10713666 10.1038/sj.onc.1203434

[12] Joerger AC, Fersht AR (2010) The tumor suppressor p53: from structures to drug discovery. Cold Spring Harb Perspect Biol 2: a000919. 20516128 10.1101/cshperspect.a000919 PMC2869527

[13] Vogelstein B, Lane D, Levine AJ (2000) Surfing the p53 network. Nature 408: 307–310. 11099028 10.1038/35042675

[14] Vousden KH, Lu X (2002) Live or let die: the cell’s response to p53. Nat Rev Cancer 2: 594–604. 12154352 10.1038/nrc864

[15] Khoo KH, Andreeva A, Fersht AR (2009) Adaptive evolution of p53 thermodynamic stability. J Mol Biol 393: 161–175. 19683006 10.1016/j.jmb.2009.08.013

[16] Klein C, Georges G, Kunkele KP, Huber R, Engh RA, et al (2001) High thermostability and lack of cooperative DNA binding distinguish the p63 core domain from the homologous tumor suppressor p53. J Biol Chem 276: 37390–37401. 11477076 10.1074/jbc.M103801200

[17] Patel S, Bui TT, Drake AF, Fraternali F, Nikolova PV (2008) The p73 DNA binding domain displays enhanced stability relative to its homologue, the tumor suppressor p53, and exhibits cooperative DNA binding. Biochemistry 47: 3235–3244. 18260640 10.1021/bi7023207

[18] Friedler A, Veprintsev DB, Hansson LO, Fersht AR (2003) Kinetic instability of p53 core domain mutants: implications for rescue by small molecules. J Biol Chem 278: 24108–24112. 12700230 10.1074/jbc.M302458200

[19] Brachmann RK, Yu K, Eby Y, Pavletich NP, Boeke JD (1998) Genetic selection of intragenic suppressor mutations that reverse the effect of common p53 cancer mutations. EMBO J 17: 1847–1859. 9524109 10.1093/emboj/17.7.1847 PMC1170532

[20] Matsumura I, Ellington AD (1999) In vitro evolution of thermostable p53 variants. Protein Sci 8: 731–740. 10211819 10.1110/ps.8.4.731 PMC2144309

[21] Nikolova PV, Henckel J, Lane DP, Fersht AR (1998) Semirational design of active tumor suppressor p53 DNA binding domain with enhanced stability. Proc Natl Acad Sci U S A 95: 14675–14680. 9843948 10.1073/pnas.95.25.14675 PMC24508

[22] Natan E, Baloglu C, Pagel K, Freund SM, Morgner N, et al (2011) Interaction of the p53 DNA-Binding Domain with Its N-Terminal Extension Modulates the Stability of the p53 Tetramer. J Mol Biol 409: 358–368. 21457718 10.1016/j.jmb.2011.03.047 PMC3176915

[23] Pantoliano MW, Petrella EC, Kwasnoski JD, Lobanov VS, Myslik J, et al (2001) High-density miniaturized thermal shift assays as a general strategy for drug discovery. J Biomol Screen 6: 429–440. 11788061 10.1177/108705710100600609

[24] Vedadi M, Niesen FH, Allali-Hassani A, Fedorov OY, Finerty PJ Jr, et al (2006) Chemical screening methods to identify ligands that promote protein stability, protein crystallization, and structure determination. Proc Natl Acad Sci U S A 103: 15835–15840. 17035505 10.1073/pnas.0605224103 PMC1595307

[25] Epps DE, Sarver RW, Rogers JM, Herberg JT, Tomich PK (2001) The ligand affinity of proteins measured by isothermal denaturation kinetics. Anal Biochem 292: 40–50. 11319816 10.1006/abio.2001.5047

[26] Sarver RW, Rogers JM, Epps DE (2002) Determination of ligand-MurB interactions by isothermal denaturation: application as a secondary assay to complement high throughput screening. J Biomol Screen 7: 21–28. 11897052 10.1177/108705710200700104

[27] Senisterra GA, Soo Hong B, Park HW, Vedadi M (2008) Application of high-throughput isothermal denaturation to assess protein stability and screen for ligands. J Biomol Screen 13: 337–342. 18448703 10.1177/1087057108317825

[28] Wang G, Fersht AR (2012) First-order rate-determining aggregation mechanism of p53 and its implications. Proc Natl Acad Sci U S A 109: 13590–13595. 22869710 10.1073/pnas.1211557109 PMC3427100

[29] Wilcken R, Wang G, Boeckler FM, Fersht AR (2012) Kinetic mechanism of p53 oncogenic mutant aggregation and its inhibition. Proc Natl Acad Sci U S A 109: 13584–13589. 22869713 10.1073/pnas.1211550109 PMC3427094

[30] Brandt T, Petrovich M, Joerger AC, Veprintsev DB (2009) Conservation of DNA-binding specificity and oligomerisation properties within the p53 family. BMC Genomics 10: 628. 20030809 10.1186/1471-2164-10-628 PMC2807882

[31] Ano Bom AP, Rangel LP, Costa DC, de Oliveira GA, Sanches D, et al (2012) Mutant p53 Aggregates into Prion-like Amyloid Oligomers and Fibrils: IMPLICATIONS FOR CANCER. J Biol Chem 287: 28152–28162. 22715097 10.1074/jbc.M112.340638 PMC3431633

[32] Ishimaru D, Andrade LR, Teixeira LS, Quesado PA, Maiolino LM, et al (2003) Fibrillar aggregates of the tumor suppressor p53 core domain. Biochemistry 42: 9022–9027. 12885235 10.1021/bi034218k

[33] Boeckler FM, Joerger AC, Jaggi G, Rutherford TJ, Veprintsev DB, et al (2008) Targeted rescue of a destabilized mutant of p53 by an in silico screened drug. Proc Natl Acad Sci U S A 105: 10360–10365. 18650397 10.1073/pnas.0805326105 PMC2492497

[34] Ishimaru D, Ano Bom AP, Lima LM, Quesado PA, Oyama MF, et al (2009) Cognate DNA stabilizes the tumor suppressor p53 and prevents misfolding and aggregation. Biochemistry 48: 6126–6135. 19505151 10.1021/bi9003028

[35] Veprintsev DB, Fersht AR (2008) Algorithm for prediction of tumour suppressor p53 affinity for binding sites in DNA. Nucleic Acids Res 36: 1589–1598. 18234719 10.1093/nar/gkm1040 PMC2275157

[36] Joerger AC, Fersht AR (2008) Structural biology of the tumor suppressor p53. Annu Rev Biochem 77: 557–582. 18410249 10.1146/annurev.biochem.77.060806.091238

[37] Dotsch V, Bernassola F, Coutandin D, Candi E, Melino G (2010) p63 and p73, the ancestors of p53. Cold Spring Harbor perspectives in biology 2: a004887. 20484388 10.1101/cshperspect.a004887 PMC2926756

[38] Moll UM, Slade N (2004) p63 and p73: roles in development and tumor formation. Mol Cancer Res 2: 371–386. 15280445

[39] Joerger AC, Allen MD, Fersht AR (2004) Crystal structure of a superstable mutant of human p53 core domain. Insights into the mechanism of rescuing oncogenic mutations. J Biol Chem 279: 1291–1296. 14534297 10.1074/jbc.M309732200

[40] Hipps DS, Packman LC, Allen MD, Fuller C, Sakaguchi K, et al (1994) The peripheral subunit-binding domain of the dihydrolipoyl acetyltransferase component of the pyruvate dehydrogenase complex of Bacillus stearothermophilus: preparation and characterization of its binding to the dihydrolipoyl dehydrogenase component. Biochem J 297 (Pt 1): 137–143. 10.1042/bj2970137 PMC1137802 8280091

[41] Joerger AC, Ang HC, Fersht AR (2006) Structural basis for understanding oncogenic p53 mutations and designing rescue drugs. Proc Natl Acad Sci U S A 103: 15056–15061. 17015838 10.1073/pnas.0607286103 PMC1635156

[42] Veprintsev DB, Freund SM, Andreeva A, Rutledge SE, Tidow H, et al (2006) Core domain interactions in full-length p53 in solution. Proc Natl Acad Sci U S A 103: 2115–2119. 16461914 10.1073/pnas.0511130103 PMC1413758

[43] Ashburner M, Thompson JN Jr (1978) The Laboratory culture of Drosophila. In: Ashburner M, Wright TRF, editors. The Genetics and Biology of Drosophila. London: Academic Press.

[44] Matthews M, Trevarrow B, Matthews J (2002) A virtual tour of the Guide for zebrafish users. Lab Anim (NY) 31: 34–40. 10.1038/5000140 11923859

[45] Hilken G, Dimigen J, Iglauer F (1995) Growth of Xenopus laevis under different laboratory rearing conditions. Lab Anim 29: 152–162. 7603001 10.1258/002367795780740276

