# Supplementary figures and images for: Stability of p53 Homologs
Source: PLoS One. 2012 Oct 24;7(10):e47889. doi: 10.1371/journal.pone.0047889 (PMC3480436; doi:10.1371/journal.pone.0047889)

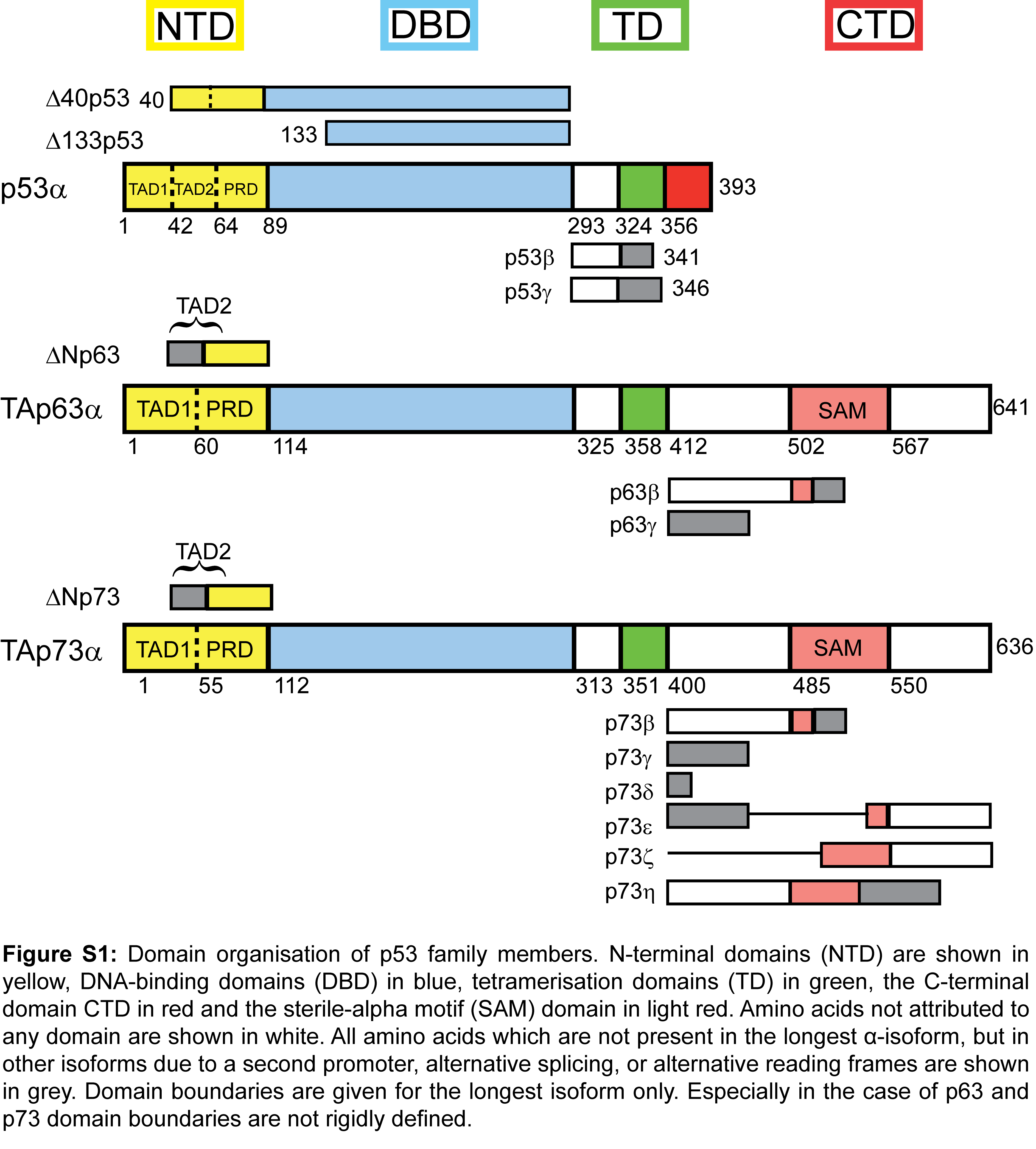

Supplement: Figure S1 — p53 family domain organisation. (TIF) [file pone.0047889.s001.tif]

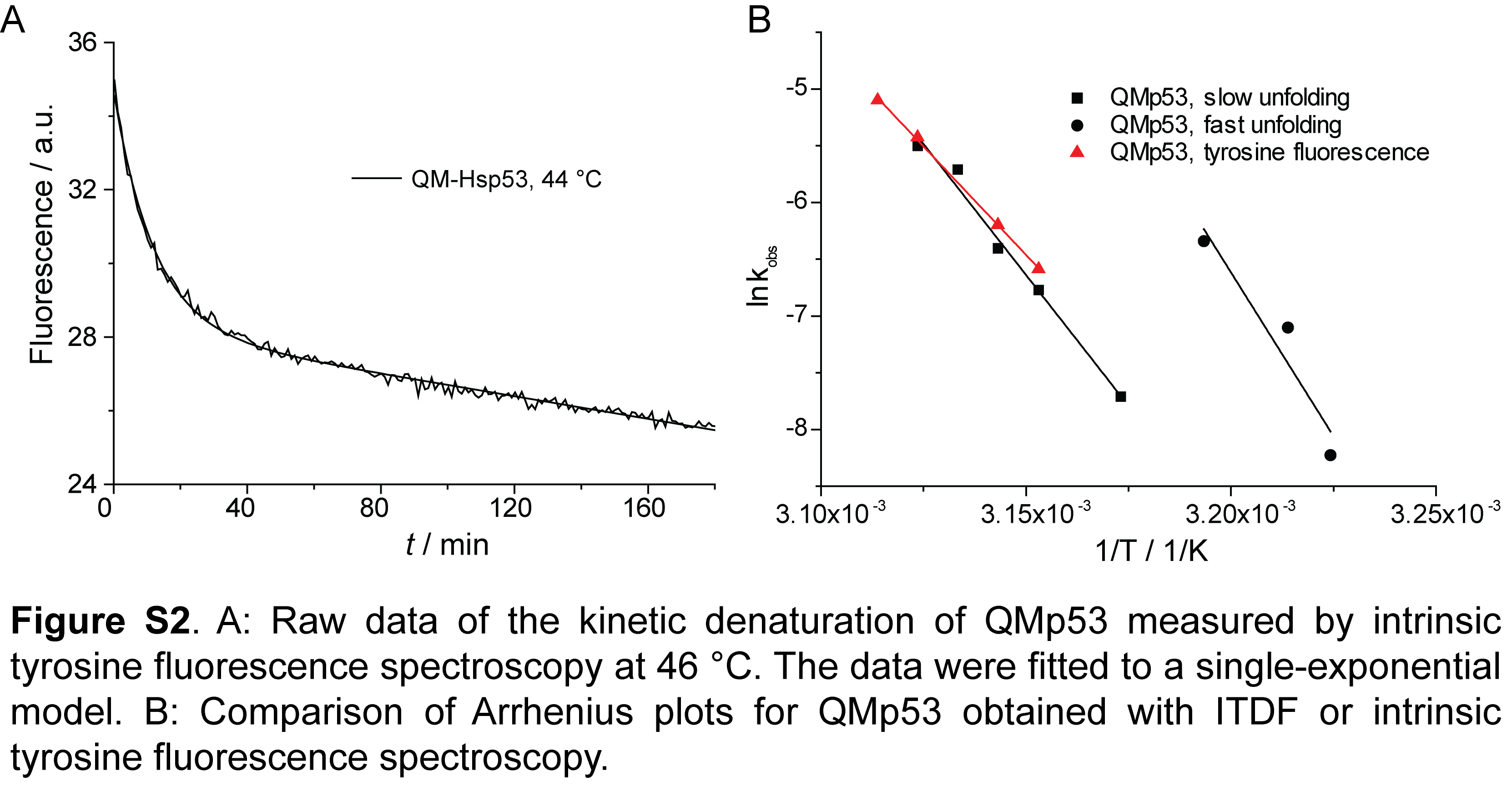

Supplement: Figure S2 — Kinetic denaturation of p53. (TIF) [file pone.0047889.s002.tif]
